# Possible active origin of replication in the double stranded extended form of the left terminus of LuIII and its implication on the replication model of the parvovirus

**DOI:** 10.1186/1743-422X-2-47

**Published:** 2005-05-31

**Authors:** Nanette Diffoot-Carlo, Lisandra Vélez-Pérez, Idaris de Jesús-Maldonado

**Affiliations:** 1 Department of Biology, University of Puerto Rico, P.O. Box 9012, Mayagüez, Puerto Rico 00680

## Abstract

**Background:**

The palindromic termini of parvoviruses have proven to play an essential role as origins of replication at different stages during the replication of their viral genome. Sequences from the left-end telomere of MVM form a functional origin on one side of the dimer replicative form intermediate. In contrast, the right-end origin can operate in its closed replicative form hairpin configuration or as a fully duplex linear sequence derived from either arm of a palindromic tetramer intermediate. To study the possibility that the LuIII left hairpin has a function in replication, comparable to that described for MVM, the replication of a minigenome containing two copies of the LuIII left terminus (LuIII Lt-Lt) was studied.

**Results:**

The data presented demonstrates that LuIII Lt-Lt was capable of replicating when NS1 helper functions were provided in trans. This extended hairpin, capable of acting as an origin of replication, lacks the arrangement of the specific domains present in the dimer duplex intermediate of MVM, the only active form of the left hairpin described for this parvovirus.

**Conclusions:**

These findings suggest that the left hairpin of LuIII has an active NS1 driven origin of replication at this terminus in the double stranded extended form. This difference between LuIII and MVM has great implications on the replication of these viruses. The presence of origins of replication at both the left and right termini in their natural hairpin form can explain the unique encapsidation pattern observed for LuIII hinting on the mechanism used by this virus for the replication of its viral genome.

## Background

Parvoviral DNA replication is a complex process that proceeds by a rolling hairpin mechanism [1-3]. Autonomous parvovirus replication and assembly occurs in the nucleus and is dependent upon host enzymes and cellular functions occurring during the S phase of the cell cycle [4-6]. MVM has been studied as a model for the replication of autonomous parvoviruses [7]. Replication initially proceeds rightward from the terminal 3' hydroxyl of the hairpin stem. The 3' hairpin serves as a primer, which allows a host polymerase to synthesize a complementary copy of the internal sequence of the viral genome until the growing strand reaches the folded back 5' terminus at the right end, resulting in a covalently closed DNA replicative form (cRF) [8]. Further processing involves the opening of the cRF at its right end by the non structural protein 1 (NS1). NS1 attaches covalently to the 5' end at the nick site via a phosphotyrosine bond [9], followed by displacement and copying of the right end hairpin, giving rise to an extended molecule designated 5' eRF [1,9,10]. Rearrangement of the copied right hand palindrome into hairpin structures creates the so-called rabbit-ear replicative form (5' reRF) [11]. This provides a primer for strand-displacement synthesis, leading to the formation of a dimer duplex intermediate (dRF) in which two unit length copies of the genome are joined by a single duplex copy of the original 3' palindrome [8,10,12,13]. In the bridge arrangement of the dRF, the mismatched doublet GA and triplet GAA are now based paired to their complementary sequences. The sequence surrounding the doublet is a potent origin, but the analogous region containing the triplet is considered completely inactive [5]. The actual sequence of the GA doublet is unimportant, but insertion of any third nucleotide here inactivates the origin, suggesting that it represents a critical spacer element [5]. The junction region thus formed contains an active NS1 driven origin [14,15].

Genetic mapping studies revealed that the minimal active MVM-3' [Genbank NC 001510] replication origin is a multi-domain structure of approximately 50 base pair (bp) sequence derived from the outboard arm of the palindromic dimer bridge structure [5,12,14]. It contains three distinct recognition elements: an NS1 binding site (ACCA)_1–3_; an NS1 nick site (CTWW↓TCA-); and a region containing a consensus activated transcription factor (ATF/CREB) binding site, essential for origin activity. NS1 binds the minimal origin in an ATP-dependent manner but is unable to initiate replication [16]. A cellular factor termed PIF, for parvovirus initiation factor, acts as an essential cofactor for NS1 in the replication initiation process allowing efficient and specific nicking of the 3' minimal origin and leaving NS1 covalently attached to the 5' end of the DNA at the nick site [16,17]. The region containing the PIF binding site is highly conserved in the 3' hairpin of other parvoviruses related to MVM, such as LuIII, H1 and MPV [16]. Once the dimer junction is formed, it is resolved asymmetrically by NS1 which introduces a single-stranded nick into the active origins generating two types of replicative form DNA: an extended palindromic form, and a turnaround form that recreates the left-hand termini [3,14,18]. The turnaround molecule generated in this way re-enters the cycle, while the extended molecule is thought to lead to the displacement of single-stranded genomic DNA, which is then packaged into pre-formed empty capsids [19].

Although the two viral telomeres are very different from each other in size, primary sequence and secondary structure, they both contain elements that become rearranged to create an NS1 dependant origin of replication, activated by different cellular cofactors. Sequences from the left-end telomere form a functional origin only on one side of the dRF intermediate [5,14]. In contrast, the right-end origin can operate in its cRF hairpin configuration and as a fully duplex linear sequence derived from either arm of a palindromic tetramer intermediate [20,21]. Unlike PIF heterocomplex, the essential cofactor for the right end origin is a non sequence- specific DNA-binding protein from the high-mobility group 1/2 (HMG 1/2) family of chromatin-associated polypeptides [20].

To study the possibility that the LuIII [Genbank M81888] left hairpin has a function in replication, comparable to that described for MVM, a minigenome containing two copies of the LuIII-3' terminus (LuIII Lt-Lt) was constructed. The sequences were cloned into the *Bam HI *site of the pUC19 vector in the head to tail-tail to head orientation, [LuIII nucleotides (nt.) 1-278/278-1]. The data presented demonstrates that LuIII Lt-Lt was capable of replicating when helper functions were provided in *trans *by pGLu883Δ*Xba*, the genomic clone of LuIII, or with pCMVNS1, an NS1 expressing vector, suggesting that this LuIII sequences contain all the *cis*-acting sequences required for excision and DNA replication. The replication of this minigenome demonstrates that the left hairpin of LuIII has an active NS1 driven origin of replication that does not have the arrangement of the dimer duplex intermediate described for MVM.

## Results and Discussion

**Figure 1:**
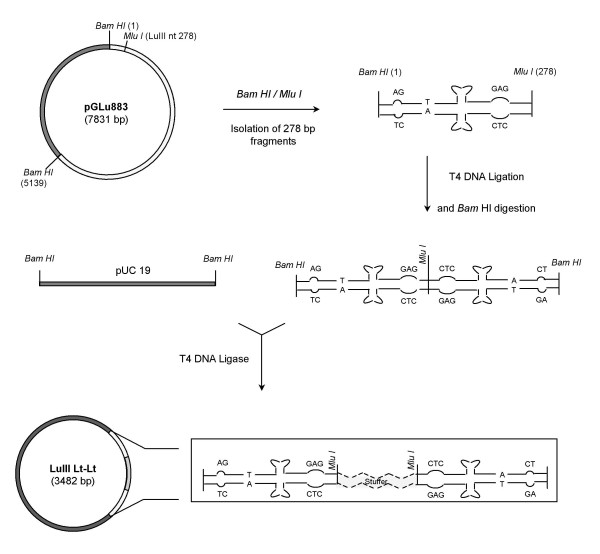
**Strategy Used to Construct LuIII Lt-Lt**. White, grey and dotted regions represent LuIII, pUC19 vector and *E. coli *sequences, respectively. Restriction enzyme sites used are indicated. PGLU883 corresponds to the LuIII infectious genomic clone.

A plasmid (LuIII Lt-Lt) containing two copies of the LuIII 3' termini flanking an *E. coli *stuffer sequence, was constructed (figure 1). In anticipation of the difficulties expected in manipulating the left end hairpin and to increase the chances of obtaining the desired construct two copies of the left end termini were successfully ligated *in vitro*, in a tail (nt 278) to head (nt 1) -head to tail orientation, this prior to cloning into pUC19. Sequencing of all recombinants obtained, with an exception, revealed a single copy of the left hairpin of LuIII ligated to *E. coli *sequences of ~250 bp. These recombinants all contained the LuIII hairpin sequence in the same orientation in pUC19 with respect to the Reverse and Forward Primers, conserving the LuIII sequence at the 5' end and the *E. coli *sequence at the 3' end. Cotmore and Tattersall [22] reported that the palindromic inserts had a greater tendency for deletions, even in recombination-deficient strains of *E. coli*, this probably due to the complex structures assumed by the inserts. Liu et al. [3] also reported inherent difficulties in cloning hairpins, resulting in many incorrect and presumably rearranged clones. The LuIII sequences may have formed a complex hairpin structure *in vivo*, due to its palindromic nature that was removed by slipped mispairing during replication [23]. Difficulty in the sequencing of these clones, particularly with the Reverse primer (M13R), supports this observation.

**Figure 2:**
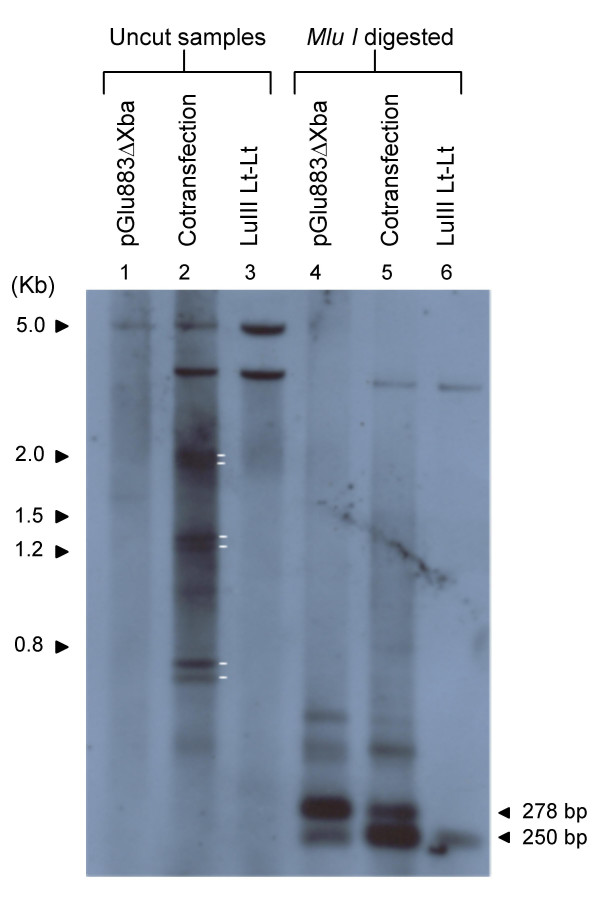
**DNA Samples Recovered From Transfection Assays of LuIII Lt-Lt Digested With *Mlu I***. Samples correspond to DNA isolated from transfection assays. Lanes 2 and 5 represent cotransfections with pGLu883Δ*Xba *and LuIII Lt-Lt. White lines indicate DNA fragments recovered from the replication of LuIII Lt-Lt. Sizes of the 2 log ladder (Roche) are shown. The probe used consisted of the insert of LuIII Lt-Lt labeled by the DNA random primed labeling method.

LuIII Lt-Lt was cotransfected with pGLu883ΔXba, the genomic clone of LuIII, by electroporation into HeLa cells. pGLu883ΔXba provides the *trans *acting factors necessary for replication of the minigenome. Southern blot analysis of the transfection assays are shown in figure 2. The blot was hybridized with the LuIII Lt-Lt *Bam HI *fragment labeled by random primed Digoxigenin-11-dUTP. Cotransfection of pGLu883ΔXba/LuIII Lt-Lt (lane 2), resulted in three sets of doublet bands. These doublets were of ~1.8, ~1.2 and ~.8 kb. These bands do not appear for the replication of the LuIII genomic clone, pGLu883ΔXba (lane 1) nor for the transfection of LuIII Lt-Lt (lane 3) for which only input plasmid was observed since the plasmid was not capable of replicating in the absence of helper functions. When DNA samples were digested with *Mlu I *(lanes 4–6) pGLu883ΔXba resulted in a strong band of ~278 bp (lane 4) corresponding to the left terminus of LuIII. Given the probe used (exclusively the LuIII Lt-Lt insert) the large fragment corresponding to nts 279-5135 of the LuIII genome was not observed on this gel. The presence of this fragment was confirmed by southern blot analysis using the full length genome of LuIII (Data not shown). Cotransfection of pGLu883ΔXba/LuIII Lt-Lt digested with *Mlu I *(lane 5) resulted in two bands, one migrating with the ~278 bp band of pGLu883ΔXba/ *Mlu I *(lane 4) and a band of greater intensity migrating slightly faster. Digestion of the cotransfection sample with *Mlu I *(lane 5) also eliminated the three sets of doublets observed in the uncut sample (lane 2) of the cotransfection suggesting that these molecules likely represent concatemers of a single molecule. Digestion of a monomer molecule resulting from the replication of LuIII Lt-Lt with *Mlu I *is expected to generate two fragments, one of ~278 bp corresponding to the left hairpin of LuIII and a band corresponding to the *E. coli *stuffer sequence which has a size of ~250 bp; two molecules of the hairpin should be generated for every copy of the stuffer sequence, therewith the intensity of the band corresponding to the hairpin is expected to be greater than the band corresponding to the stuffer sequence. Two bands were observed for this digestion (lane 5); the larger band migrates along side the band observed for pGLu883ΔXba likely representing the left end hairpin of LuIII in double stranded form. The smaller of the two bands, of greater intensity, may represent the left hairpin with an alternate conformation. A faint band of similar migration is observed for pGLu883ΔXba when digested with *Mlu I *(lane 4). The band corresponding to the stuffer sequence is not obvious, this likely due to its similar migration to the LuIII left end with a different conformation. Lane 6, containing the transfection sample of only LuIII Lt-Lt shows a band of ~250 bp resulting from the digestion of input plasmid that was not capable of replicating, this confirms our assumption that the stuffer sequence observed in lane 6 migrates similar to the left hairpin with an altered conformation hence its greater intensity when compared to the migration of the double stranded left hairpin.

**Figure 3:**
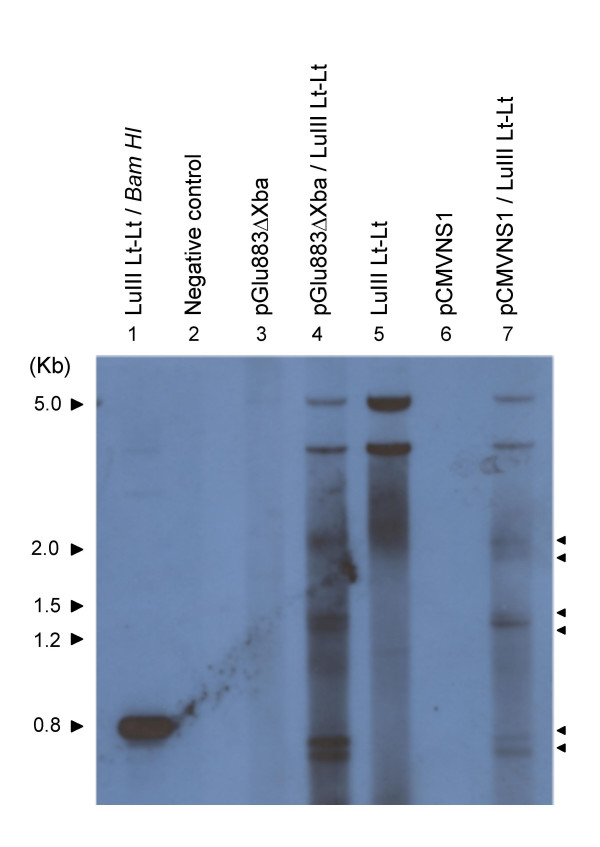
**DNA Recovered from Transfection Assays of LuIII Lt-Lt with pCMVNS1**. DNA samples shown correspond to: 1. the full length insert isolated from LuIII Lt-Lt, 2. negative control of transfection, 3–7. DNA isolated from transfection assays of the indicated samples. Arrow heads point to DNA fragments recovered from the replication of LuIII Lt-Lt. Sizes of the 2 log ladder (Roche) are indicated. The probe used consisted of the insert of LuIII Lt-Lt labeled by the DNA random primed labeling method.

LuIII Lt-Lt was also cotranfected with pCMVNS1, an expression vector for the MVM nonstructural protein NS1 (figure 3). LuIII Lt-Lt was capable of replicating when only NS1 was present in *trans *(lane 7) resulting in the same banding pattern as observed in figure 2 (lane2). It has been suggested that the non-structural protein NS1 makes the excision [4] by introducing a single-stranded nick, possibly at the 5' end of the viral genome. If the minigenome could replicate under these conditions, it contains all the *cis*-acting sequences required for excision and DNA replication. These results suggest that LuIII Lt-Lt was capable of excision and replication when pGLu883ΔXba or pCMVNS1 was provided in *trans *and that only NS1 viral functions appear to be required for the excision and replication of LuIII Lt-Lt.

**Figure 4:**
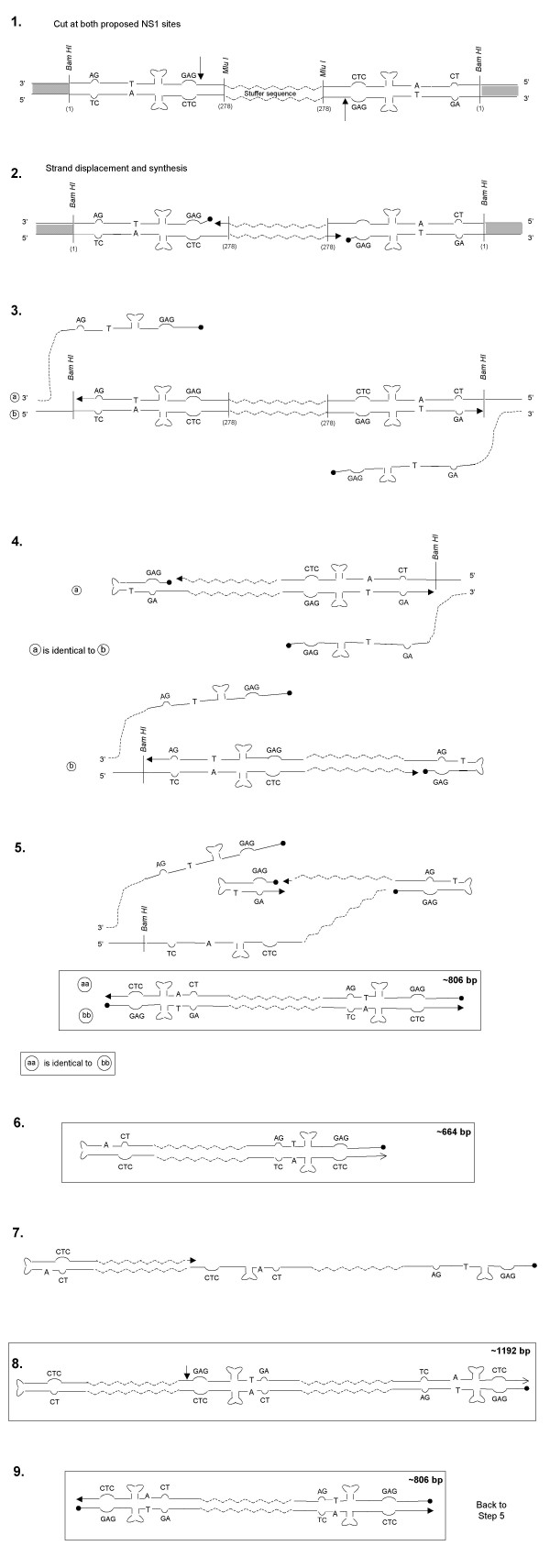
**Proposed Model for the Rescue and Replication of LuIII Lt-Lt**. Restriction sites and their positions with respect to the LuIII sequence are indicated. Grey, white and zigzag regions represent pUC19, LuIII left terminus and *E. coli *sequences respectively. In steps 4 and 5 the molecules generated (a/b and aa/bb) are identical, for this reason only one molecule at each step is continued throughout the scheme. The estimated sizes of some of the molecules (boxed) are indicated.

A possible mechanism for the replication of LuIII Lt-Lt is shown in figure 4. The model proposes a nick at the NS1 nick site present at the left hairpin (step 1); this generates two origins of replication running in opposite directions (step 2) that lead to strand displacement. The new hairpins assume secondary structures and continue DNA synthesis (step 4), generating a close-end molecule. This step generates two copies of a molecule estimated to be ~664 nts in length. Both molecules can now generate a monomer length molecule of ~806 bp (step 5). As a result of replication, the arrangement of the arms in the hairpin change resulting in hairpins with the GAG triplet present at the 5'end of the molecules. This forces the molecule to go through a dimer intermediate (steps 7 and 8) generating a molecule with a turn around end of ~1192 nts in length. This dimer is then resolved to generate monomer length double stranded molecules (step 9). The sizes of the DNA molecules obtained from this model on the replication of LuIII Lt-Lt correspond very closely with the sizes of the products predicted by the model (figure 2 and 3) for the replication of LuIII Lt-Lt.

Parvovirus DNA replication starts when the 3' hydroxyl group at the left end of the viral genome primes the synthesis of a complementary strand, leading the formation of a double stranded replicative form known as the cRF. *In vitro *studies have shown that the cRF of autonomous parvovirus like MVM terminates in closed hairpins at both ends, making cRF a major, or even obligatory intermediate of parvovirus replication [8], but only the right-end hairpin is resolved in the presence of NS1 [8,24]. The cRF is re-opened and copied, producing a right end extended form (5' eRF) followed by unfolding of the hairpin and copying of the terminal sequence. This leads to the formation of dimeric RF (dRF) and higher-order concatamers that would be resolved into monomeric (mRF) RF DNA. If the wild type LuIII virus replicates using the mechanism described for MVM and forms the cRF, the replication of two copies of the left end such as in LuIII Lt-Lt should result in a dead molecule that could not be resolved by NS1. Although the terminal palindromic sequences are essential for the replication of the APVs genome, the right and left terminal sequences are not equivalent in function [25,26]. According to the modified rolling hairpin model of MVM replication, the right end origin is active in the covalently closed hairpin configuration and also in the extended right end telomere [14,24]. In contrast, the MVM left end inverted repeat does not constitute a replication origin in the hairpin configuration and needs to be copied in the form of a left-to-left end bridge to be subsequently resolved at the multimeric RF DNA stage [1,3,8,14,27].

**Figure 5:**
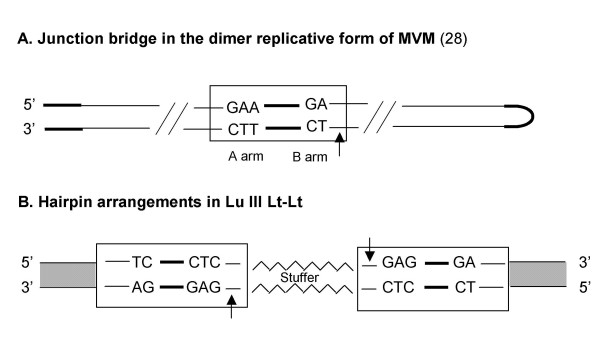
**Comparison of the MVM Dimer Bridge (A) with the Hairpin Arrangement in LuIII Lt-Lt (B)**. Hairpins and NS1 recognition nick sites are indicated by dark bold lines and arrows respectively. The grey patterned boxes correspond to pUC19 sequences.

When the dimer bridge origin of MVM is compared to the left end arrangement in LuIII Lt-Lt (figure 5), it becomes apparent that the left terminus is an incomplete origin of replication based on the origin proposed for MVM replication. A competent replication origin contains, among other things an NS1 nick site. If like MVM, the left end terminus of LuIII is only processed when present as a bridge in the dimer RF but not as a hairpin in monomeric replicative form, neither of the left end termini in LuIII Lt-Lt would be recognized by NS1. As a result, the LuIII insert would not be excised from the plasmid pUC19, and hence no replication would be expected to occur. Comparison of the sequences present in LuIII Lt-Lt with the junction bridge in the dimer replicative form of MVM [28] (figure 4) illustrates that the A and B arms of the LuIII left end are organized differently from that proposed for the active origin of replication for MVM. Unlike the dimer arrangement described for MVM, in LuIII Lt-Lt the CT doublet is positioned at the 5'end and the CTC triplet is positioned inboard at the 3'end in both hairpins. In the hairpin arrangement an NS1 nick site is not present at the 5' end of the CT bubble as described in the MVM dimer bridge. Nevertheless, LuIII Lt-Lt was capable of replication suggesting that the left hairpin of LuIII does constitute a replication origin in the extended double stranded hairpin configuration.

**Figure 6:**
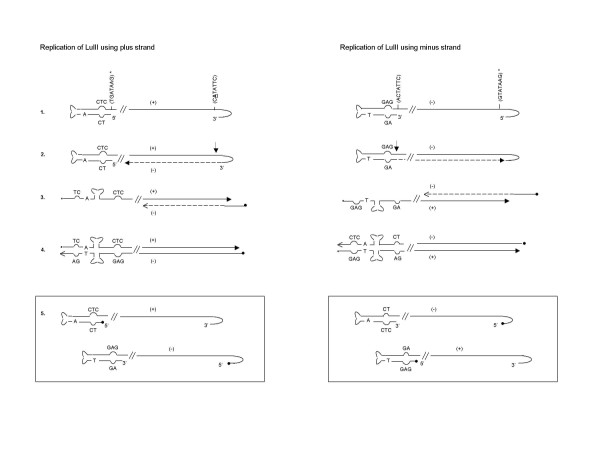
**Proposed Model for the Replication of Parvovirus LuIII**. A model for the replication of the (+) and the (-) strand of LuIII is shown. The NS1 nick site and its complementary sequence (*) are indicated. The unpaired sequences present at the left hairpin are shown. The arrows point to NS1 nick sites. A corresponds to the insertion in the NS1 nick site present at the right terminus of LuIII.

Given the functionality of the left hairpin of LuIII as an origin of replication in the extended double stranded form a replication model of LuIII can be predicted resulting in equivalent amounts of plus and minus DNA viral strands (figure 6). In this model the plus and minus DNA strands, independently initiate replication from the right and left hairpins respectively (step 1). The NS1 nick sites present at the left and right termini in LuIII differ from each other; there is an insertion of an Adenine residue in the NS1 nick site present at the 5' terminus of LuIII. This additional adenine is also not present in the NS1 nick site described for MVM [29].

This replication model for LuIII predicts flip/flop conformations at both termini. Earlier studies [30] in which the left and right termini of the minus and plus strands, respectively, were labeled at the 3' hydroxyl group and subsequently digested with *Hha I *suggested that the left terminus of the LuIII minus strand exists only in the flip conformation, and the right terminus of the plus strand exists in both the flip and flop conformations. Numerous bands were observed when the left terminus of the minus strand was digested with *Hha I *yet these were justified as alternate secondary structures of the hairpin in the flip conformation. The expected fragments for the digestion of the flip and flop conformations of the left hairpin are very similar in size, any slight variation in migration due to the secondary structures assumed by these fragments could have impaired the interpretation of the results. The conformation present at the left end of the plus strand still remains unknown.

## Conclusion

The data presented demonstrates that LuIII Lt-Lt contains all the *cis*-acting sequences required for excision and DNA replication when NS1 viral functions are provided in *trans*. These findings suggest that the left hairpin of LuIII has an active NS1 driven origin of replication at this terminus in the double stranded extended form. This extended hairpin, capable of acting as an origin of replication, lacks the arrangement of the specific domains present in the dimer duplex intermediate of MVM, the only active form of the left hairpin described for MVM. This difference between LuIII and MVM has great implications on the replication of these viruses. The presence of origins of replication at both the left and right termini can explain the unique encapsidation pattern observed for LuIII hinting on the mechanism used by LuIII for the replication of its viral genome.

## Methods

### Construction of LuIII Lt-Lt

The LuIII Lt-Lt minigenome (figure 1) has two copies of the left end palindrome of the autonomous parvovirus LuIII (nt. 1-278) cloned into the *Bam HI *site of pUC19 [29] [Genbank L09137]. The 3' hairpin of LuIII was obtained from pGLu883 [30], the full-length genomic clone of LuIII cloned into the pUC19 vector. pGLu883 was digested with both *Bam HI *(pUC19 nt. 417) and *Mlu I I *(LuIII nt. 278) for two hours at 37°C and then electrophoresed on a 1.2% agarose gel in 1X TBE buffer at 75 V. The *Bam HI */ *Mlu I I *digestion generated three fragments of approximately 278, 2686, and 4861 bp. The 278 bp fragment corresponding to the left end hairpin was isolated and purified using the Geneclean Spin Kit^® ^(QBio-gene, Carlsbad, CA), and then were ligated through the *Mlu I *site in an overnight reaction at 4°C using 1 U of T4 DNA ligase. The ligation was digested with *Bam HI *generating a fragment of 568 bp corresponding to the two copies of the 3' hairpin in a "head to tail-tail to head" conformation (nts 1-278, 278-1). The fragment generated was purified as described and ligated into the *Bam HI *site of pUC19 that was previously treated with calf intestinal alkaline phosphatase (CIAP) (Roche Applied Science, Indianapolis, IN) for one hour at 37°C.

### Preparation of Competent Cells

Two different strains of Escherichia coli were used as competent cells: DH5α [(lacZ.M15. (lacZYA-argF) recA1 endA1 hsdR17 (rkmk+) phoA supE44 thi gyrA96 relA1)] (ATCC, Rockville, MD) and SURE^®^2 super competent cells [(e14- (McrA-). (mcrCB-hsd SMR-mrr) 171 endA1 supE44 thi-1 gyrA96 relA1 lac recB recJ sbcC umuC::Tn5 (Kanr) uvrC (F' proAB lacIqZ.M15 Tn10 (Tetr) Amy Camr)] (Stratagene, La Jolla, CA). Competent cells were prepared by the calcium chloride method [31].

### Transformation of Competent Cells

The recombinant molecules were transformed in both DH5α and SURE^®^2 competent cells. Competent cells were thawed on ice for 15 minutes (min.). The DNA was added to the cells and incubated on ice for 30 min. Cells were heat-shocked in a 42°C water bath and subsequently incubated on ice for 2 min. DH5α and SURE^®^2 competent cells were heat-shocked for 2 min. and 30 seconds respectively. 100 μL of preheated (42°C) LB broth was added to both cell samples and incubated at 37°C for 1 hour (h) with shaking at 225 rpm. DH5α transformed cells were spread on LB agar plates containing 50 mg/mL ampicillin and 80 μL of 2% X-gal. SURE^®^2 transformed cells were spread on LB plates containing 50 mg/mL ampicillin, 100 μL of 2% X-gal and 100 μL of 10 mM IPTG.

### Isolation of DNA Recombinants

The resultant plasmids from DH5α and SURE^®^2 transformed cells were purified by the alkaline lysis miniprep method, described by Ausubel et al. [31] and analyzed with restriction enzymes. Sequencing was performed at the New Jersey Medical School, Molecular Resource Facility.

### Tissue Culture

HeLa (ATCC, Rockville, MD) cells were grown in Minimal Essential Medium (MEM Eagle) (MP Biomedicals, Aurora, OH) supplemented with 10% fetal bovine serum (FBS) (HyClone, Logan, UT) and PSG (8 mM Penicillin G, 3 mM Streptomycin Sulfate, 200 mM L-Glutamine). They were incubated at 37°C in 25 and/or 75 cm^2 ^plastic tissue culture flasks. For sub-culturing, the cells were rinsed twice with Phosphate-Buffered Saline (1X PBS) and incubated in 1X Trypsin (Difco, Detroit, MI) for 5 min. at 37°C. Cells were harvested by centrifugation at 3800 rpm for 5 min. at 4°C. The resultant pellet was resuspended in the medium described above and seeded into culture flasks at a proportion of 1:3.

### Transfection Assay

HeLa cells were grown to 100 % confluency in a 75 cm^2 ^flask. They were washed three times with 1X PBS and then tripsinized at 37°C for 5 minutes. Cells were harvested by centrifugation at 3,800 rpm for 5 min. at 4°C and washed in 10 ml of PBS. Cells were resuspended and split at a proportion of 1:9. Approximately, 5 μg of pGLu883ΔXba, LuIII Lt-Lt minigenome and pCMVNS1 were added to the corresponding tubes and incubated at 37°C for 10 min. Cells were transferred to sterile cuvettes with a 4-mm gap width, and electroporated individually at 230 V and 950 μF using a capacitance discharge machine (Gene Pulser, Bio-Rad Laboratories, Hercules CA). After each pulse, 700 μL of MEM-10% FBS were added to the cuvette and the cells were resuspended carefully. The electroporated cells were incubated for 45 min. at 37°C and then transferred to 25 cm^2 ^flasks containing 3 mL MEM-10% FBS. After an overnight incubation at 37°C, the medium was changed, and the cells were incubated at 37 °C until the low molecular weight DNAs were isolated at five days post-transfection, as described by Tam and Astell [25]. DNA samples were resuspended in 30 μL TE (10 mM Tris-HCl, 1 mM EDTA, pH 8.0).

### Southern Blot Analysis

Samples were electrophoresed on a 1.2% agarose gel in 1X TAE buffer at 80 V, and passively transferred onto a Zeta Probe nylon membrane (Bio-Rad Laboratories, Hercules, California) as described by Ausubel et al [31]. Probes were labelled by the random primed DNA labeling method with Digoxigenin-11-dUTP (Roche Applied Science, Indianapolis, IN). The blot was hybridized at 50°C and washed at 55°C. Detection was performed according to manufacturer's instructions (Roche Applied Science, Indianapolis, IN).

## Competing interests

The author(s) declare that they have no competing interests

## Authors' contributions

**NDC **drafted and revised critically the manuscript, had the intellectual idea of the study and its design, contributed significantly in the analysis and interpretation of the data, proposed the replication models presented and gave the final approval of the version to be published.

**LVP **constructed LuIII Lt-Lt, collected the data resulting from the transfection of LuIII Lt-Lt/pGluΔXba, contributed in the analysis and interpretation of the data, participated in the idea and design of the models proposed and in the drafting and revision of the manuscript.

**IDM **collected the data resulting from the transfections of LuIII Lt-Lt/pGluΔXba and, LuIII Lt-Lt/pCMVNS1, contributed in the analysis and interpretation of the data, participated in the design of the models proposed and in the drafting and revision of the manuscript.

All authors read and approved the final manuscript.
